# The effectiveness of problem solving therapy in deprived South African communities: results from a pilot study

**DOI:** 10.1186/1471-244X-11-156

**Published:** 2011-09-30

**Authors:** Edith van't Hof, Dan J Stein, Isaac Marks, Mark Tomlinson, Pim Cuijpers

**Affiliations:** 1 Department of Psychiatry and Mental Health, University of Cape Town, Cape Town, South Africa; 2 Department of Clinical Psychology, VU University Amsterdam, Amsterdam, The Netherlands; 3 Institute of Psychiatry, King's College London, London, UK; 4 Department of Psychology, University of Stellenbosch, Stellenbosch, South Africa

## Abstract

**Background:**

The majority of South Africans with a DSM-IV diagnosis receive no treatment for their mental health problems. There is a move to simplify treatment for common mental disorders (CMDs) in order to ease access. Brief problem solving therapy (PST) might fill the treatment gap for CMD's in deprived communities in South Africa. This pilot study evaluates the feasibility, acceptability and effectiveness of this PST program for CMD's in deprived communities around Cape Town.

**Methods:**

A Dutch problem solving program was adapted and translated into English, Xhosa and Afrikaans and thereafter implemented in townships around Cape Town. An initial attempt to recruit participants for online PST proved difficult, and so the program was adapted to a booklet format. Volunteers experiencing psychological distress were invited to participate in the either individually or group delivered 5-week during self-help program. To evaluate the effectiveness, psychological distress was administered through self-report questionnaires. After completion of the intervention participants also rated the program on various acceptability aspects.

**Results:**

Of 103 participants, 73 completed 5 weeks of brief PST in a booklet/workshop format. There were significantly more dropouts in those who used the booklet individually than in the group. Psychological distress measured on the K-10 and SRQ fell significantly and the program was evaluated positively.

**Conclusions:**

The results suggest that brief problem solving in a booklet/workshop format may be an effective, feasible and acceptable short-term treatment for people with CMD's in deprived communities. In this setting, group delivery of PST had lower drop-out rates than individual delivery, and was more feasible and acceptable. Randomized controlled trials are needed to evaluate the effect of brief self-help PST more rigorously.

## Background

Common mental disorders are very prevalent and significantly impact the lives of many people in low-and middle-income countries, including South Africa. A nationally representative study revealed that 30% of South African adults had a DSM-IV disorder in their lifetime, including 16% with an anxiety disorder and 10% with a mood disorder [1]. Depression and anxiety disorders can be chronic [2], severely impair quality of life [3], cause excess mortality [4], and incur substantial societal costs [5]. In South Africa, psychiatric disorders were ranked as the third most disabling condition, after HIV/AIDS and other infectious diseases [6], while many HIV/AIDS sufferers had comorbid mental disorders [7].

Despite this high prevalence of common mental disorders, many who suffer from these conditions do not receive treatment. In a recent survey, only a quarter of South Africans with a 12-month DSM-IV diagnosis of anxiety or depression had been treated in the preceding year [8,9]. This is unsurprising as there are many barriers to treatment, including the fact that resources are inadequate and unevenly distributed across the country [9-13]. Most services are located in the urban areas and are still characterized by patterns created by racial segregation and inequities during the apartheid system [14,15]. The integration of mental health care into primary care facilities has been said to lack behind due to primary care nurses' large workloads and lack in mental health training [16].

The number of people with mental health problems receiving no treatment is 30-50% in high income countries and 76-80% in low-and middle income countries (LAMICs) [17]. The World Health Organization (WHO) notes that this treatment gap is mainly due to a scarcity of human, mental health and financial resources which are also caused by policies in LAMIC. Many people who seek help are not treated with evidence-based interventions [18]. Socio-economic status determines access to facilities, creating a huge inequity in access to mental health care in most low and middle-income countries [19]. The WHO has called for increased investment in mental health research, particularly in LAMIC [20] and the scaling up of services [21]. Research can reinforce the commitment of policymakers and provide a concrete evidence-based programme to upscale care for mental disorders and to reduce the treatment gap for mental disorders in LAMIC.

Researchers are currently investigating the development of easily accessible, evidence-based, and cost-effective treatments. Self-help problem solving therapy (PST) could be one such approach. Guided-self help has had promising efficacy in anxiety disorders [22-24], unipolar depression [24-27], alcohol addiction [28], sexual dysfunction [29], weight loss [29], and phobias [30]. PST self-help can be guided face to face or by book, phone, interactive voice response, CD-ROM, television, video or the Internet. PST is a cognitive behavioural therapy (CBT) technique which is applicable in primary care [31] and cost-effective in treating common mental health problems [32-37]. It assumes that depression and anxiety symptoms are often caused by practical everyday problems and aims to teach people better ways to cope with such problems by setting goals and minimizing feelings of incompetence and distress [38]. PST can be sensitive to local needs, is efficacious for depression [32-37] and anxiety and emotional disorders [39,40], is well-established in developed world settings and requires fairly limited resources.

PST is thus a good candidate to narrow the treatment gap for mental disorders in South Africa. However, data on psychotherapies from high-income countries may not be generalisable to LAMICs [41]. First, cultural factors can influence the conceptualization of common mental disorders by both patient and care providers. The attitudes of people in developed and developing countries often differ regarding the kind of help needed to resolve a disorder. Explanatory models in many low and middle-income countries may be less likely to acknowledge psychobiological factors in psychological distress. This may affect attitudes regarding the kind of help needed and the acceptability of mental health interventions. Second, the infrastructure of health systems across countries can diverge extensively. The applicability of treatment research from developed to LAMIC countries can be influenced by the scarcity of mental health manpower, the growth of the private medical sector, rising health costs, and changing health care financing systems [41].

This pilot study aimed to adapt and test the feasibility and acceptability of low-cost PST in South African communities which have little or no access to mental health services. Our original implementation used a web-guided format of PST in different deprived communities around Cape Town. We found that the lack of access to a computer and the Internet and the lack of basic computer and Internet literacy among community members probably played a role in the poor take-up of our web-guided program, making it difficult to implement a web-guided intervention. When the recruitment of online PST users proved difficult we adapted the PST to a booklet format to see if this was more feasible and acceptable. Why online PST wasn't feasible and acceptable in South African communities is discussed in more detail in a previous paper [42]. After deliberation with local community workers, we converted the online PST content to a booklet-plus-workshop PST version and evaluated its use. This paper presents the pilot results using the booklet and workshop version in deprived Cape Town communities.

## Methods

### Development of the intervention

The Dutch PST (Problem Solving Therapy) website 'Allesondercontrole.nl' was translated into English, Xhosa and Afrikaans, the three official languages spoken in South Africa's Western Cape Province. The accuracy of the translations was checked by back-translating the English version (called 'Taking control') and Afrikaans version (called 'Vat beheer') into Dutch and the Xhosa version (called 'Lawula ubomi bakho') into English. A team of experts adapted the case vignettes to make them relevant to our population by identifying three themes of major relevance: HIV/AIDS, unemployment, and trauma due to violence. Three comprehensive clinical vignettes were constructed around these site-specific topics and were reviewed by South African experts in psychology, psychiatry and social work.

### Recruitment of participants

After fieldwork and visits to several communities, we offered booklet PST in various communities with limited access to mental health care. Recruitment was by: posting pamphlets and posters on notice boards of community libraries, community centres and public health facilities and by giving short talks on the program in community settings. These recruitment methods drew very few participants, so we worked with local non-governmental organisations (NGOs) to refer people to our program. The NGOs gathered people to attend short talks about the project by the project manager and assistants in English, Xhosa or Afrikaans.

We recruited people from 4 different communities representing deprived communities around Cape Town with lack of access to mental health care. The first, Langa, is a black township with mostly formal housing which was established in 1923. Khayeltisha, another black township, is a much younger, urbanizing, mainly post-apartheid township whose housing is mostly informal. The third is a coloured township named Manenberg - created by the apartheid government in 1966 and 1970 for low-income coloured families. It has mixed formal and informal housing. In order to study a rural community as well, we included Mamre - 60 km from Cape Town - which was founded as a Moravian mission station in 1808 and is a rural village with housing mainly consisting of small cottages.

Volunteers at each site were invited to participate if they felt they had mental health problems. They were excluded if they: 1. had suicide plans, rather than just suicidal thoughts; 2. had severe mental health problems - such patients were referred to their local community day hospital, in line with the Western Cape's mental-health-referral guidelines; 3. were too illiterate to read the booklet's self-help text in English, Afrikaans or Xhosa.

### The Intervention

An initial attempt to recruit participants for online PST proved difficult, and so the program was adapted to a booklet format. The adapted Dutch self-help Problem Solving Therapy called "Alles onder controle" ('Taking control') is based on the self-examination therapy of Bowen et all [43]. In ''self-examination therapy'' subjects learn to take control of their problems and their lives by (1) determining what really matters to them, (2) investing energy solely in problems related to what matters, (3) thinking less negatively about the problems which are unrelated, and (4) accepting situations that cannot be changed. It was developed as a self-help booklet which proved efficacious in several studies [43,44].

'Taking control' consists of three steps: 1. describe what really matters to you in life; 2. write down current worries and problems and sort them into three categories: (a) unimportant problems (unrelated to things that matter), (b) soluble problems, and (c) insoluble problems (e.g., the loss of a loved one). For unimportant problems techniques to cope with negative thoughts are suggested (worry time, thought stopping, log of positive thoughts). Insoluble problems are addressed by explaining the natural course of coping processes and tips on how to speed acceptance (e.g., accepting negative feelings, talking with other people and establishing contact with others in a similar situation). Soluble problems are addressed in six steps: i) describe the problem, ii) brain-storm solutions, iii) choose the best solution, iv) make a plan for carrying out the solution, v) actually carry out that solution, vi) evaluate the results. Coping with traumatic experiences is managed by explaining techniques to help the coping process (putting into words, impact statement, work through emotions, reconnect). In the last (3^rd^) step, subjects are asked to draw up a plan for the future describing how they would try to accomplish the things that matter most to them. The booklet also contains vignettes of people using the PST techniques to solve their problems and their homework assignments over five weekly lessons.

### Format of the intervention

The pilot study tested 'Taking control' as a booklet. Subjects in the individual format were asked to work through the booklet on their own for 5 weeks by reading the weekly materials. Over the five weeks they were offered weekly brief phone support by trained coaches in their own language. The coaches were a psychologist (author EH) and a clinical psychology graduate working on the project, both trained by author PC. The coaches used a structured one-page instruction template to guide participants through the process, and noted the call's content and its duration. Participants could text a free 'please call me' message to their coach saying when they could receive the weekly support phone call.

Due to a high drop-out rate with individual therapy, difficulties reaching participants, and needs expressed by participants, we tried instead to deliver the 'Taking Control' book to volunteers in *groups *which met weekly for 45-60 minutes over 5 weeks. Each group had 6-15 members. During group sessions members were given the next week's chapter of 'Taking control' and were guided regarding problems and homework difficulties encountered with the previous chapter. Members were asked to read, between sessions, the relevant part of the book and complete its associated homework assignments. In group meetings members shared their experiences on how to deal with problems and worries. They could get the same phone support between sessions as subjects who used the book individually.

Whether people used the book individually or in a group depended on time of enrolment in the study and accessibility to group meetings. After the switch to delivering the book in groups most new volunteers opted the group format, though subjects still had the choice of going through the book individually. When assigning people to groups no active matching took place for language, cultural background or gender. Since the groups mostly contained people from a specific community participant were very similar in cultural background and language.

### Data collection

Prior to the intervention, participants completed a questionnaire concerning demographic variables (age, gender, education, marital status, occupation, ethnic background, number of children, first language, religion, living location) and current mental health problems. The Kessler Psychological Distress Scale (K-10), Self Reporting Questionnaire for mental health symptoms (SRQ) and the Boston University Empowerment Scale (BUES) were administered before session 1 and after session 5 to evaluate the effect on mental health symptoms. The K-10, SRQ and BUES were translated from English into Xhosa and Afrikaans by a back-translation process. After session 5 participants also rated 'Taking Control' for acceptability, satisfaction, credibility, length and format, ease of reading, and content. The 'acceptability and feasibility questionnaire' was developed by the team to elicit attitudes of the participants towards specific elements of the intervention. Participants had to indicate their level of agreement on 12 statements about the intervention (as reported in Table 1) using a 5 point likert scale. The last 4 questions of the questionnaire evaluate the participants' perception of helpfulness of different group and content aspects of the intervention (as reported in Table 2).

**Table 1:** Evaluation of the program; percentage of people in different answer categories

Response range (1-5)	Strongly disagree	Disagree	Undecided	Agree	Strongly agree	Response average
Language used is understandable	-	-	22	24	53	4.3

Book is easy to read		2	8	44	45	4.3

Stories apply to my problem	2	6	29	34	29	3.8

Case stories are useful	1	6	7	31	56	4.3

Homework was useful	-	2	9	52	37	4.2

Advice on solving problems helped a lot		1	8	50	42	4.3

Advice on solving problems apply to my problems	1	6	33	28	33	3.9

Advice on 3 problems was useful	1	1	13	44	40	4.2

Book helped me to overcome depression/anxiety	2	2	8	28	60	4.4

I would recommend this book	1	1	2	36	60	4.5

Total respondents						90

**Table 2:** Percentages of people agreeing and disagreeing on the helpfulness of specific components of the intervention

N = 27	% Yes	% No
**Content treatment**:		

Learning ways to cope with negative feelings	82	18

Learning to solve problems in 6 steps	67	33

Learning ways to cope with trauma	52	48

Learning ways to cope with loss or life changing event	60	40

**Group dynamics**		

Feeling understood by others	60	40

Chance to help others	52	48

Feeling accepted and valued	70	30

Creating hope by seeing other people overcome problems	67	34

### Data analyses

The differences in pre and post scores were evaluated by t-test. We obtained maximum-likelihood estimators for our missing data using the Expectation-Maximization (EM) algorithm, and did t-tests after imputing missing data. We used χ^2 ^statistics to evaluate associations between categorical variables and an ANCOVA to detect changes in effect for individually- and group- delivered 'Taking Control'. Finally, we did Wilcoxon testing of the difference between the number of people falling into various K-10 categories before and after the intervention.

## Results

### Participants

A total of 103 subjects participated in the study, 57 in its individual format and 46 in its group format. The mean age of people who enrolled in the PST program was 37; 22% were male; 29% were black Africans and 71% coloured (mixed race). Most lived in Manenberg (58%) and Khayelitsha (21%), 90% were unemployed and 46% were married. Regarding education, 6% had finished higher education, 22% had finished high school, and 33% had not finished primary school. When asked what kind of mental health problems were present the most commonly mentioned were: being stressed (72%), worrying a lot (29%), being depressed (23%), being sad (16%) and thinking too much (12%). The baseline K10 scores indicated that 9% of our participants scored low, 22% moderate, 43% high and 26% very high. (Table 3)

**Table 3:** Pre-post test differences K10 categories

N = 73	Pre %	Post %	p-value
**Low **(1-15) *may currently not be experiencing significant feelings of distress.*	8.9	31.5	0.001

**Moderate **(16-21) *experiencing mild levels of distress consistent with a mild depression or anxiety disorder*	22.8	31.5	0.131

**High **(22-30) *experiencing moderate levels of distress consistent with a moderate depression or anxiety disorder*	42.6	26	0.011

**Very high **(30-50) *experiencing severe levels of distress consistent with a severe depression or anxiety disorder*	25.7	11	0.005

Of the 103 participants, 73 completed 'Taking Control'. Completion was not significantly associated with sex, marital status, age, employment or education. There were significantly more dropouts in those who used the book individually (44%, n = 25/57) than in the group (11%, n = 5/46); χ^2 ^(1) = 13.4, p < .001; (OR = 6.5, 95% CI: 2.2-18.6). There was no difference in age with between people who finished and dropped out (t (98) = 0.14, p > .05.

There was a significant association between dropout rate and living location and both living location (χ^2 ^(2) = 6.53, p < .05) and ethnic group (χ^2 ^(3) = 12.235, p < .01). The odds of people dropping out were higher for people living in Khayelitsha than in Manenberg (OR = 2.9, Cl: 0.94 - 8.81). The odds of people dropping out was higher for people from a Xhosa than Coloured background (OR = 1.8, Cl: 0.66 - 5.1).

### Effectiveness of the intervention

Analyses of just completers found that mental health symptoms fell significantly from before to after using 'Taking Control' on both the K10 (pre: M = 20.33, SE = 7.14; post: M = 25,79, SE = 7.48, *t *(72) = 6.87, p < 0.05, d = 0.63) and on the SRQ (pre: M = 5.59, SE = 4.77; post: M = 7.25, SE = 4.93, *t *(31)= 2.041, p = 0.05, d = 0.34). Participants reported being significantly more empowered on the BUES post (M = 50.21, SE = 5.28) than pre-participation (M = 47.86, SE = 4.67), *t *(36) = -2.361, p < 0.05, d = 0.43 (Table 4). Intent-to-treat analyses after imputing missing data did not reveal different results (Table 4). An ANCOVA showed no significant effect of individual versus group format of delivery on the post K-10 scores after controlling for the K10 pre scores F(1,70) = 2,26, p = 0.137. Table 3 shows the number of participants in each K10 category, with an obvious reduction in percentages in the high and very high categories after the intervention. There was a significant decrease in participants in the 'very high' category (p = 0.005), and the 'high' category (p = 0.011). The number of participants in the 'low' category increased significantly (p < 0.001) and in the 'mild' group tended to increase (p = 0.131).

**Table 4:** Pre-post differences on measures from analyses on completers only and imputed data analyses

Completers only							
	**Pre**		**Post**		**Comparison pre-post**		

	**Mean**	***SE***	**Mean**	***SE***	**Mean Drop**	**Effect size**	**p<**

**K-10** n = 73	25.79	*7.48*	20.33	*7.14*	5.46	0.63	0.001

**SRQ** n = 32	7.25	*4.93*	5.59	*4.77*	1.66	0.34	0.05

**BUES** n = 37	47.86	*4.67*	50.13	*5.28*	-2.27	0.43	0.024

**Intent to treat**							

**K-10** n = 103	25.34	*7.5*	20.08	*6.40*	5.26	0.66	0.001

**SRQ** n = 49	7.16	*4.01*	5.69	*3.95*	1.47	0.37	0.001

**BUES** n = 49	48.36	*5.55*	50.23	*4.78*	-1.87	0.31	0.025

### Evaluation of program

Halfway through recruitment the high drop-out rate (44%), difficulties in reaching participants and feedback on their individual use of 'Taking Control' showed a need to switch to its use in groups. Difficulties in reaching participants on cell phones also made it harder to give weekly coaching support by phone. Many preferred face-to-face support. A strong preference for sessions in groups were expressed by participants going through 'Taking Control' individually and by people attending recruitment talks for our project. To meet the needs of those receiving treatment we therefore switched to trying out the group format.

The aspects of 'Taking Control' which participants regarded as most helpful were 'learning to cope with negative feelings' (82%), 'feeling accepted and valued' (70%), 'learning to solve problems in six steps' (67%) and 'creating hope by seeing other people overcome problems' (67%). Table 2 shows the percentages of 'yes' and 'no' answers on the remaining questions. The percentages of people feeling helped 'a lot' or 'an enormous amount' were 73% for 'being part of a group' and 64% for 'the content of the intervention'.

The great majority of the participants rated the 'Taking Control' booklet as easy to read (89%) and its language understandable (77%) (Table 1). Many found the case studies (78%) and homework assignments (89%) useful components of the program. 'Taking Control''s focus on problem solving was rated as helpful by 92% of participants and the distinction between 3 different types of problems was said to be useful by 84%. A large majority of participants said the course helped them with their feelings of depression, distress and anxiety (88%) and almost all would recommend the course to others (96%). However, a substantial number felt that the examples did not show how to solve problems (39%) and the case studies portrayed (37%) were not applicable to their problems. Suggestions for which problems deserved more guidance within 'Taking Control' were; not being excepted in the community due to homosexuality, and drug abuse and violence at home.

## Discussion

This pilot studied the feasibility, acceptability and effectiveness of 'Taking Control', a booklet guidied brief short-term problem solving therapy (PST) for people with mental health problems in deprived communities around Cape Town. In these communities the approach was feasible and acceptable. We found a higher drop-out in the individual format than in the group delivery format. Mental health symptoms improved significantly over the 5 weeks of the PST. Participants' sense of empowerment increased as they adopted solutions suited to the local context and skills to enhance control of their lives.

In our communities group delivery of PST had lower drop-out rates than individual delivery, and was more feasible and acceptable. Participants said non-specific group factors like altruism (chance to help others), universality (feeling understood and similar to others) and cohesion (feeling accepted and valued) were as helpful as the content of the book. We did not, however, find a significant difference in improvements between group and individual delivery of 'Taking Control' in our pilot. Non-specific factors like therapeutic alliance, providing a rationale for the symptoms and healing setting have an effect on both group and individual formats of the intervention. Group interventions have additional non specific factors, which could influence the effect of an intervention. Previous findings indicate that specific as well as non-specific therapy factors can yield similar improvement [45,46]. More research is needed to identify and evaluate the actual active components in PST programmes. This will contribute to the identification of potential components to focus on in strengthening the intervention.

Of course our pilot study has the limitations of a small sample size, absence of a control group, and non-random assignment to group or individual use of the 'Taking Control' booklet. The improvement and sense of empowerment might have resulted from spontaneous remission or placebo effects. After 5 weeks of participation there was no further follow up to check sustainability of the results.

In a recent Indian study individually delivered brief problem solving was not more effective than placebo for people with common mental disorders [47]. The authors considered that insufficient structure of the therapy and the fact that their primary care patients thought and expected that they should receive medication might have been reasons for finding no effect. Another problem was poor adherence to their problem solving interventions. The problem solving sessions were delivered individually and took place in clinics, which differs from our use of group session in community settings. The poor adherence rate might be an indication for low acceptability of the treatment in the research population and another reason for finding no effect. They authors remark that many problems people face in low and middle income countries are embedded in social problems so a purely clinical program may be insufficient to bring relief [48].

The results of our pilot fit with a growing literature on mental health care in low and middle income countries attempting to determine the best mode and type of mental health treatment in these settings. An increasing number of studies show the potential of group interventions for mental disorders in LAMICs. Bolton *et al *found that group interpersonal psychotherapy was feasible and effective in treating depressive symptoms in Ugandan adults and adolescents [49-51]. A Chilean psycho-educational group intervention was effective in reducing depressive symptoms in women [52]. In Iran, group CBT was superior to fluoxetine in treating depression and anxiety of infertile women [53]. In addition, group based interventions were effective in controlling birth outcomes and maternal depression in India and Nepal [54,55]. An often mentioned possible advantage of group over individual treatment is cost-effectiveness. A recent review did not, however, find evidence of group CBT being more cost-effective than individual CBT [56]. Group psychotherapy was cost-effective in the LAMIC Uganda [57] and a primary stepped-care program for depressed women in Chile was significantly more effective and only slightly more expensive than care as usual [58].

There are, however, still relatively few studies showing the feasibility and efficacy of group interventions in LAMIC. Many cultural, political, economic and social differences may limit the generalizability of these studies. More randomised controlled trials are needed of group versus individual delivery in such low-income communities, and stepped care programs. Although the studies conducted had promising results, the acceptability of group interventions in many LAMICs is still unclear. Traditionally many find it inappropriate to discuss personal details with people outside the family, and this could complicate the use of such group of treatments [48]. The feasibility and cost-effectiveness of group PST should be studied by implementing the intervention in primary care facilities with lay workers as group facilitators. In this way the cost can be minimized and be compared to the care as usual costs made when people present themselves with CMD's in primary care clinics.

The encouraging results of the present pilot study suggest that it is worth studying further applications of group PST to reduce the burden of common mental disorders in deprived communities. The intervention was sensitive to local needs, fostered active coping styles and allowed for indigenous solutions suited to the local cultural context. However, some adaptations should be considered in future testing of our 'Taking Control' program. More group sessions might be needed after particularly severe or numerous traumas. Locally prominent problems such as drug abuse, not being accepted in the community due to homosexuality, and violence need to be addressed in more detail. Our coaches were a trained psychologist and a psychology student, but PST conducted by non-professionals has been effective [36,40]. So further work is needed to see if PST can be task-shifted away from a psychologist and to trained lay people, making it more feasible to integrate in primary health care or other consisting care structures. The high prevalence of mental disorders in deprived communities emphasizes the potential value of low-cost interventions and task-shifting.

## Conclusion

The results suggest that brief problem solving in a booklet/workshop format may be an effective, feasible and acceptable short-term treatment for people with CMD's in deprived communities. In this setting, group delivery of PST had lower drop-out rates than individual delivery, and was more feasible and acceptable. Randomized controlled trials are, however, needed to test the efficacy and cost-effectiveness of group PST more rigorously and to justify its future scaling up in LAMIC.

## Competing interests

The authors declare that they have no competing interests.

## Authors' contributions

All authors contributed to the study design. EH carried out the data collection. EH, PC and DS undertook the data-analyses. EH wrote the first draft of the manuscript with authors PC, DS, IM and MT contributing to data interpretation and providing critical reading of the manuscript. All authors contributed to and have approved the final manuscript.

## Pre-publication history

The pre-publication history for this paper can be accessed here:

http://www.biomedcentral.com/1471-244X/11/156/prepub
